# How data knowledge transformation affects teachers' precision teaching ability: The mediating effect of data consciousness

**DOI:** 10.3389/fpsyg.2022.1076013

**Published:** 2022-12-07

**Authors:** Jia Tao, Chenchen Fan, Wenwen Wu, Yongyuan Zhu

**Affiliations:** ^1^School of Educational Science, Anhui Normal University, Wuhu, Anhui, China; ^2^Party and Government Office, Anhui Medical University, Hefei, Anhui, China

**Keywords:** data knowledge, teachers' precision teaching ability, mediating effect, knowledge transformation, data consciousness

## Abstract

With the development of smart education, teachers' precision teaching ability has increasingly become an important component of their professional ability. Based on the Socialization, Externalization, Combination, Internalization (SECI) model of knowledge management, this research constructs a theoretical model of data knowledge transformation, data consciousness, and teachers' precision teaching ability, and uses Partial Least Squares to analyze the interaction and influence among the elements. The empirical results show that the transformation of internal knowledge related to data has a significant positive impact on the improvement of teachers' precision teaching ability and data consciousness; the transformation of external knowledge related to teaching has a positive effect on the teaching management ability of teachers' precision teaching ability, but the effect is not significant. Data consciousness plays a partial mediating effect between internal knowledge transformation and teachers' precision teaching ability, and a complete mediating effect between external knowledge transformation and teaching management ability.

## Introduction

Precision teaching is a representative of technology-enabled education in the era of big data, and is a major structural change in the field of teaching and learning, which brings many new challenges to the academia (Ministry of Education of the People's Republic of China, 2021). The incongruity and mismatch between teachers' previous teaching experience and data knowledge transformation is one of the challenges teachers face in promoting precision teaching (Cui and Zhang, 2022). Some studies have reported that teachers' data knowledge transformation implies teachers' ability to recognize, use, and evaluate data technology (Mandinach and Gummer, 2013). Teachers who have better data knowledge transformation skills are more successful in implementing precision teaching (Cabero et al., 2022). It can be argued that teachers' lack of attention to or misconceptions about the development of data knowledge transformation skills can directly affect the improvement of teachers' precision teaching skills. To examine exactly how data knowledge transformation affects teachers' precision teaching ability, we first need to understand what teachers' precision teaching ability is.

Research on teachers' precision teaching ability reveals that scholars have affirmed the importance of precision teaching, while also raising a series of questions during the inquiry. Hayes et al. (2018) noted that precision teaching realizes the automation of teaching but weakens the pedagogical significance of teachers' experience. Fletcher et al. (2013) take the smart campus as the context and reveal the current increased workload of teachers in precision teaching, such as the diversification of roles and the refinement of division of functions. Kubina and Yurich (2009) verified that the practice of precision teaching is positively related to teachers' personal ability to use data through precision teaching research. Moreover, some scholars investigated the definition of teachers' precision teaching ability. For example, Peng and Zhu (2018) proposed that teachers' precision teaching ability allows them to use learning data to enhance teaching, propose teaching strategies based on evidence, and implement teaching practice. Pierce et al. (2014) argued that to achieve precision teaching, teachers should have professional (statistics) and situational (data generation) knowledge. Theall (2001) argued that teachers' precision teaching ability is a high-level ability to gather practical teaching experience and data analysis skills, and to learn scientific theories. However, Mandinach (2012) posited that teachers' precision teaching ability is basically consistent with their data literacy; Mandinach proposes that teachers' precision teaching ability is a comprehensive ability that can transform data into teaching strategies to meet students' specific needs. Scholars have differing views on the definition of teachers' precision teaching ability.

Data consciousness is a concept at the cognitive level, which requires teachers to know that data is meaningful and valuable, and be aware of the possible harm caused by poor data management. From the existing studies, there seems to be a lack of empirical research on the relationship between data knowledge transformation, teachers' precision teaching ability and data consciousness. Therefore, this study aims to verify the mediating effect model of teachers' data knowledge transformation, data consciousness, and precision teaching ability through questionnaire data under the framework of knowledge management theory, and provide evidence to clarify the mechanism of action among the three. The research questions of this study are as follows:

- (RQ1)Does teachers' data knowledge learning and transformation have an effect on the improvement of their precision teaching ability? How does it affect?
- (RQ2)What is the role of teachers' data consciousness in the relationship between data knowledge transformation and teachers' precision teaching ability?

## Literature review and theoretical assumptions

Analyzed from the perspective of pedagogy, the teaching practice mainly contains four dimensions: learning situation analysis, teaching management, teaching decision, and teaching evaluation (Srikoom et al., 2018). Therefore, teachers' precision teaching ability can be seen as a comprehensive ability of teachers to use data to advance the four dimensions of learning analysis, teaching management, teaching decision making, and teaching evaluation. From the perspective of the socialization, externalization, combination, internalization (SECI) model of knowledge management, the improvement of teachers' precision teaching ability mainly includes explicit knowledge and implicit knowledge, explicit knowledge is directly related to data and can be shared in a standardized and systematic language, implicit knowledge mainly includes data applications, understanding, data-driven teaching and learning, and other knowledge related to beliefs, metaphors, and thinking patterns (Revuelta-Domínguez et al., 2022). These elements have interacted and changed together during the transformation of explicit and implicit knowledge to create a whole knowledge innovation. This process of the externalization, integration, internalization, and socialization of knowledge ultimately points to the improvement of teachers' precision teaching ability (Weinberger and Green, 2022).

### The relationship between data knowledge transformation and teachers' precision teaching ability

The transformation of data knowledge corresponds to teachers' precision teaching ability. Based on the SECI model, teachers' data knowledge can be divided into two categories. One is internal knowledge transformation, which focuses on teachers' learning of data-related technologies and resources; the other is external knowledge transformation, which focuses on the combination of teacher experience and data knowledge, and runs through the processes of teaching management, evaluation, decision-making, and others.

#### Internal knowledge transformation and teachers' precision teaching ability

Explicit knowledge of data mainly includes development and settings of the platform, reading and calculation of the data, use and processing of software, and data security (Duraiswami and Krishnamurthy, 2020), The absorption of internal knowledge mainly comes from thematic learning. Before teachers use technical means to carry out precision teaching, they need to learn a lot of relevant data knowledge, especially dealing with technology. Research has found that the effects of precision teaching are often limited by teachers' mastery of platforms, software, and other data tools (Lyons, 1984). The accumulation of teachers' internal knowledge can effectively improve their understanding of precision teaching, reduce the costs of trial and error, and play an important role in teachers' active precision teaching changes (Wang et al., 2011). Therefore, this paper asserts that the transformation of internal knowledge can effectively promote the improvement of teachers' precision teaching ability, and we propose the following hypotheses:

*H1: There is a positive correlation between internal knowledge transformation and teachers' precision teaching ability*.
*H1a: There is a positive correlation between internal knowledge transformation and student analysis ability*.
*H1b: There is a positive correlation between internal knowledge transformation and teaching management ability*.
*H1c: There is a positive correlation between internal knowledge transformation and educational decision-making ability*.
*H1d: There is a positive correlation between internal knowledge transformation and instructional evaluation ability*.

#### External knowledge transformation and teachers' precision teaching ability

Tacit data knowledge mainly includes data analysis and evaluation, educational decision-making and integration, and conclusion dissemination, among others (Kabir and Carayannis, 2013). In the process of introducing external knowledge, teachers actively participate in data processing, which can improve their ability to engage in data-driven teaching. While absorbing external knowledge, teachers can improve their own educational and teaching experience through creation, exchange, integration, and other activities. In the application stage of external knowledge, teachers promote the sharing of new knowledge generated by precision teaching practice to recreate new knowledge and promote the personalized reform of education. This process is the ultimate goal of precision teaching (Haigh, 2008). Therefore, this paper posits that the transformation of external knowledge has a positive impact on improving teachers' precision teaching ability and promoting precision teaching practice. We offer the following hypotheses:

H2: There is a positive correlation between external knowledge transformation and teachers' precision teaching ability.
*H2a: There is a positive correlation between external knowledge transformation and student analysis ability*.
*H2b: There is a positive correlation between external knowledge transformation and teaching management ability*.
*H2c: There is a positive correlation between external knowledge transformation and educational decision-making ability*.
*H2d: There is a positive correlation between external knowledge transformation and instructional evaluation ability*.

### The relationship between data knowledge transformation and data consciousness

#### Internal knowledge transformation and data consciousness

According to existing research, teachers can have clear conflicts with the data generated in teaching (Du and Su, 2021). There are three main reasons for such conflicts. First, it is difficult to correctly interpret different forms of measurement data; second, it is difficult to convert existing information into appropriate teaching strategies to solve the problem of data representation; and third, it is difficult to interpret large data sets, resulting in a certain sense of powerlessness and fear. This sense of teaching burden generated by the data is one of the major challenges to the in-depth practice of precision teaching at this stage. Internal knowledge related to data can be learned through centralized learning, communication, text training, and other explicit tasks (Friedman et al., 1979). Therefore, this paper asserts that through the study of internal knowledge, such as data, teachers' fears of data can be effectively alleviated to deepen their knowledge and understanding of data, which has a positive impact on the cultivation of teachers' data consciousness. Therefore, the following hypothesis is proposed:

H3: There is a positive correlation between internal knowledge transformation and data consciousness.

#### External knowledge transformation and data consciousness

For teachers, learning about knowledge related to educational data is meaningless if it is separated from the educational context. However, the results of our survey show that at this stage, the internal knowledge learning of relevant data is mainly controlled by administrative departments, such as education authorities and schools, which characterized by policy and compulsion. We also note that after learning about these topics, teachers hardly use data knowledge; the reason for this is that teachers lack understanding of the value of data (Williamson, 2012). Training for precision teaching focuses on the internal knowledge level, and teachers equate this with technology learning, without paying much attention to it. In the learning and transformation of external knowledge, strengthening deduction skills, exchange, data sharing, and data-driven education practice can help teachers realize the advantages of data-driven precision teaching. Therefore, this paper argues that the transformation of external knowledge also has a positive impact on improving teachers' data consciousness. The following hypothesis is proposed:

H4: There is a positive correlation between external knowledge transformation and data consciousness.

### The relationship between data consciousness and teachers' precision teaching ability

From the research on the professional quality of pedagogical students' education and teachers' post-service education, data-related knowledge is not the focus of training, according to the Professional Standards for Teachers in Kindergartens, Primary Schools, and Middle Schools, which were customized by the Ministry of Education in 2012. Rather, the professional quality of teachers in China mainly includes three aspects: teacher ethics, professional knowledge, and professional ability (Ministry of Education of the People's Republic of China, 2012). Based on the current general trend of smart education development, data-driven decision-making has gradually been integrated into all aspects of teachers' ethics, professional knowledge, and professional ability requirements. Teachers often face difficulties in learning and transforming data-related knowledge before applying this knowledge in precision teaching practice. In the current training program, although teachers have learned new data knowledge through professional development, they still know little about data comprehension, technology, and new content (McDowall et al., 2021). From the learning of data knowledge to learning how to apply data in teaching and learning, teachers need to awaken their personal interest in data learning. Therefore, this paper suggests that the cultivation of teachers' data consciousness has a positive impact on improving teachers' precision teaching ability, and the following hypotheses are proposed:

H5: There is a positive correlation between data consciousness and teachers' precision teaching ability.
H5a: There is a positive correlation between data consciousness and student analysis ability.
H5b: There is a positive correlation between data consciousness and teaching management ability.
H5c: There is a positive correlation between data consciousness and educational decision-making ability.
H5d: There is a positive correlation between data consciousness and instructional evaluation ability.

### The mediating role of data consciousness

As internal and external knowledge are transformed, teachers have changed focus from the learning of explicit statistical knowledge to the implicit formation of an individual understanding of data. The latter has in turn transformed into information through internalization and been applied to teaching practice to guide the processes of student analysis, teaching management, educational decision-making, and instructional evaluation. The transformation of explicit and implicit knowledge is a two-way, cyclical accumulation process (Wieser, 2016). Individual teachers acquire the ability and teaching experience working with data and statistics through learning or practice, guided by data consciousness; the educational significance of the raw data is identified through the process of connection and the internalization of explicit knowledge. The teacher's movement from understanding data to summarizing information and decision-making in teaching practice is the process of discovering practical problems, creating new knowledge, assimilating existing implicit knowledge, and innovating and integrating to generate new explicit knowledge under the impetus of data consciousness. Whether it is the transformation of internal knowledge or the transformation of external knowledge, this process ultimately points to the improvement of teachers' precision teaching ability.

Knowledge is a component of data consciousness (Filderman et al., 2022), and data consciousness is the cornerstone of cultivating teachers' precision teaching ability. Therefore, this paper argues that data consciousness plays a mediating role between internal and external knowledge transformation and teachers' precision teaching ability. We propose the following hypotheses:

H6: Data consciousness plays a mediating role between data knowledge and teachers' precision teaching ability.
H6a: Data consciousness plays a mediating role between internal knowledge and student analysis ability.
H6b: Data consciousness plays a mediating role between internal knowledge and teaching management ability.
H6c: Data consciousness plays a mediating role between internal knowledge and educational decision-making ability.
H6d: Data consciousness plays a mediating role between internal knowledge and instructional evaluation ability.
H6e: Data consciousness plays a mediating role between external knowledge and student analysis ability.
H6f: Data consciousness plays a mediating role between external knowledge and teaching management ability.
H6g: Data consciousness plays a mediating role between external knowledge and educational decision-making ability.
H6h: Data consciousness plays a mediating role between external knowledge and instructional evaluation ability.

**Figure 1:**
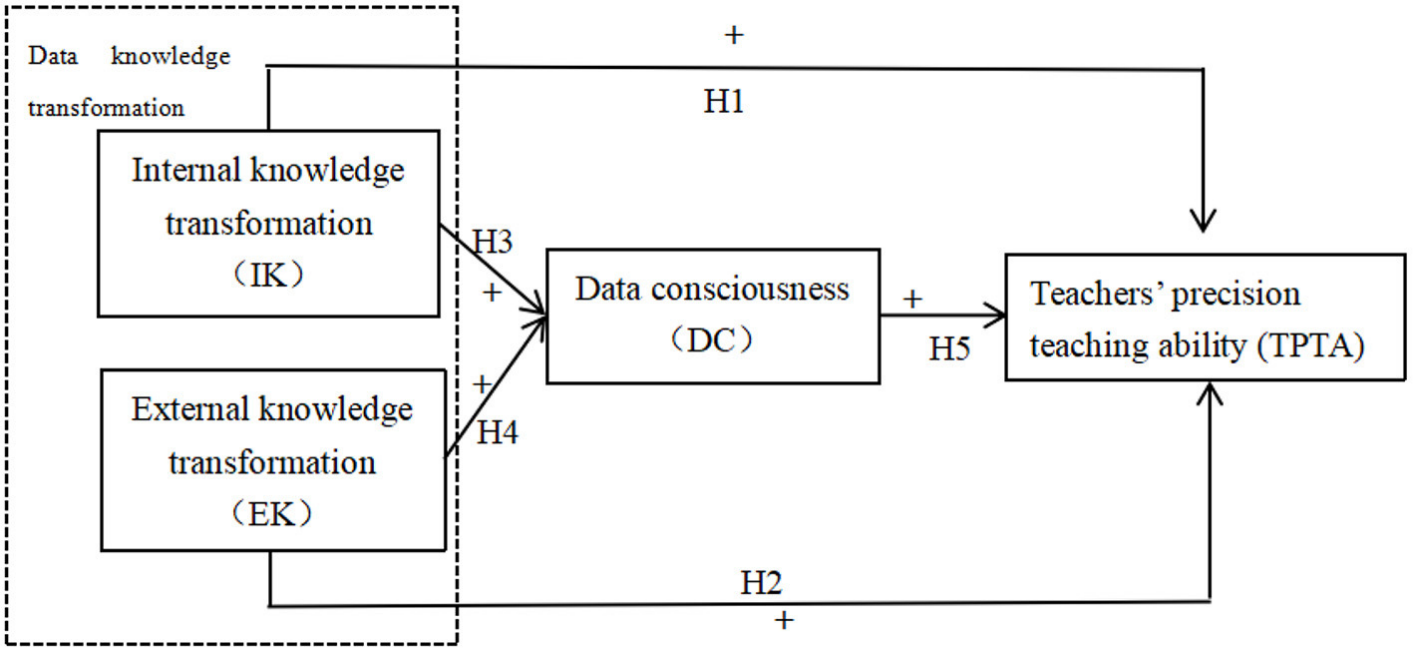
A theoretical model of data knowledge transformation, data awareness, and teachers' precision teaching ability.

On the basis of related research and the above theoretical discussion, we propose the following theoretical model (Figure 1).

## Research method

### The survey instrument

The scales that measure data consciousness were developed by the research team based on a series of publications by Chinese scholars. The scales comprise three dimensions, which were informed by the theoretical assumptions and research results of the extant literature. Moreover, on the basis of the existing research dimensions, the measures provide response options on a five-point Likert scale, as shown in Table 1.

**Table 1:** Reference source and items of measuring scale.

**Scale dimension**			**Item**	**References**
**First-level dimensions**	**Second-level dimensions**	**Third-level dimensions**		
Internal knowledge transformation		Data positioning	4	Zhang and Li, 2015
		Data acquisition		
		Data analysis		
		Data interpretation		
External knowledge transformation		Find problems	4	Ruan and Zheng, 2016
		Make educational decision		
		Monitor teaching development		
		Reflect and evaluate		
Teachers' precision teaching ability	Student analysis ability	Sensitivity	4	Chen, 2012
		Reason		
		Thinking		
		Culture		
Teachers' precision teaching ability	Teaching management ability	Ability to manage teachers	3	Liu, 2006
		Ability to manage students		
		Ability to manage teaching environment		
	Educational decision-making ability	Teaching plan	3	Song and Li, 2011
		Teaching interaction		
		Teaching behavior		
	Instructional evaluation ability	Evaluation method	3	Zhao, 2003
		Evaluation content		
		Evaluation function		
Data consciousness		Student analysis	4	Liu et al., 2021
		Teaching management		
		Educational decision-making		
		Teaching evaluation		

After developing the scale, the measures were repeatedly revised and discussed to ensure understanding, accuracy, and the representativeness of specific items. Ten front-line teachers who have participated in the knowledge training for the precision teaching system were invited to conduct a pre-test of the questionnaire to check question wording and the order of specific items. Case descriptions were added to the questions about the dimensions of teachers' precision teaching ability to help respondents understand the specific aspects of their ability. In addition, before the questionnaire was administered, we confirmed that the teachers participating in the survey understood the differences between internal knowledge transformation and external knowledge transformation. We also added instructions to the questionnaire guidelines.

### Participants

The participants in this study are front-line teachers who have participated in smart education, precision teaching projects, competitions, training, and other activities. Respondents are from Shanghai, Anhui, Zhejiang, Jiangsu, Guangdong, and other provinces. The survey was administered from June 2021 to August 2021. First, 98 questionnaires were distributed (and all were recovered; recovery and effective rates were both 100%) with the help of the characteristics of the school and its major, and with the help of the interpersonal and working relationships of graduates and teachers. Second, we entrusted the principals of certain cooperative schools to distribute 150 questionnaires (of which 147 were recovered); the recovery rate is 98% and the effective rate is 96.7%. In total, 243 valid questionnaires were collected from teachers at 45 schools, including 6-year primary schools, 3-year junior high schools, 3-year high schools, 9-year consistent schools, and 6-year full middle schools. Among them, more than 60% of the respondents had more than 10 years of work experience, and 73% of the respondents had participated in the training and carried out precision teaching practice.

### Analysis on reliability and validity of the survey instrument

We used IBM SPSS version 25 to analyze the reliability and validity of the questionnaire to ensure its accuracy.

#### Reliability analysis

To test the reliability of the generated items and the scale, Cronbach's α is used for detection; the test results are shown in Table 2. When values are >0.8, the scale is considered acceptable. The questionnaire is divided into four categories: internal knowledge transformation, external knowledge transformation, teachers' precision teaching ability, and data consciousness. The reliability of each sub-scale is 0.918, 0.926, 0.964, and 0.872, respectively. Because these values are higher than 0.8, the reliability is considered high. Therefore, reliability analysis can be conducted.

**Table 2:** Scales and Cronbach's α values.

**Scale**	**Cronbach's α**
Internal knowledge transformation	0.918
External knowledge transformation	0.926
Teachers' precision teaching ability	0.964
Data consciousness	0.872

To ensure that the data obtained from the questionnaire are suitable for a factor analysis, the KMO (Kaiser-Meyer-Olkin) and Bartlett tests were conducted on the sub-scales and the total scale. When KMO values are larger than 0.7 and approach 1, the partial correlation between variables is strong, and factor analysis is more accurate. When the significance level of Bartlett's sphericity test is <0.05 and the correlation between variables is strong, factor analysis can be conducted. The test results are shown in Table 3. The KMO values of the sub-scales and the total scale were >0.7, and the *p*-values of the Bartlett test was <0.05; thus, this questionnaire is suitable for factor analysis (Liang et al., 2020).

**Table 3:** KMO and Bartlett test.

**Scale**		**Data knowledge transformation**	**Teachers' precision teaching ability**	**Data consciousness**	**Whole scale**
KMO		0.942	0.947	0.768	0.959
Bartlett test of sphericity	Approximate chi square	1,860.923	3,084.682	681.121	8,075.281
	Degrees of freedom	28	66	10	406
	*p*-value	0.000	0.000	0.000	0.000

KMO, Kaiser-Meyer-Olkin.

#### Validity analysis

The study conducted a CFA (Confirmatory Factor Analysis) on seven factors and 24 analysis items. The effective sample capacity was 243, or 10 times more than the number of analysis items. This level is moderate, so the data can undergo convergence validity and discriminant validity analyses. The factor loading of items reflects the correlations between the factors and analysis items. When the standard load coefficient is >0.7, there is a strong correlation between factors and analysis items. As shown in Table 4, the factor loading coefficients of items are >0.7. The composite reliability (CR) and average variance extracted (AVE), can comprehensively characterize the convergent validity of the scale. Generally, when the CR value is >0.7 and the AVE is >0.5, the convergence validity of the scale is high. As shown in Table 4, the AVE values corresponding to the seven factors are all >0.5, and the CR values are all higher than 0.7, indicating that the analysis and data have good convergence validity.

**Table 4:** Results of confirmatory factor analysis.

**Latent variable**	**Measurement items**	**Factor loading**	**CR**	**AVE**
Internal knowledge transformation	IK1	0.850	0.918	0.736
	IK2	0.847		
	IK3	0.859		
	IK4	0.874		
External knowledge transformation	EK1	0.877	0.927	0.760
	EK2	0.876		
	EK3	0.851		
	EK4	0.882		
Student analysis ability	SA1	0.848	0.885	0.658
	SA2	0.857		
	SA3	0.777		
Teaching management ability	TM1	0.758	0.909	0.769
	TM2	0.916		
	TM3	0.819		
Educational decision-making ability	ED1	0.893	0.919	0.792
	ED2	0.880		
	ED3	0.908		
Instructional evaluation ability	IE1	0.882	0.883	0.717
	IE2	0.931		
	IE3	0.836		
Data consciousness	DC1	0.766	0.839	0.639
	DC2	0.886		
	DC3	0.85		
	DC4	0.639		

CR, composite reliability; AVE, average variance extracted; IK, internal knowledge; EK, external knowledge; SA, student analysis ability; TM, teaching management ability; ED, educational decision-making ability; IE, instructional evaluation ability; DC, data consciousness.

CFA can be used to distinguish validity results. The diagonal lines in Table 4 are AVE square root values, and the other cells are correlation coefficients. The value of the square root of the AVE represents the convergence of factors, and the correlation coefficient represents the correlation between items. If the factor has strong convergence (i.e., obviously stronger than the absolute value of the correlation coefficient with other factors), it can indicate that it has discriminant validity. The results are shown in Table 5. The square root of the AVE of each factor is greater than the absolute value of the correlation coefficient of this factor and other factors; therefore, all factors have good discriminant validity.

**Table 5:** Distinguishing validity: Pearson correlations and AVE square root.

	**IK**	**EK**	**SA**	**TM**	**ED**	**IE**	**DC**
IK	0.858						
EK	0.791	0.871					
SA	0.783	0.766	0.812				
TM	0.728	0.779	0.608	0.876			
ED	0.699	0.618	0.587	0.595	0.890		
IE	0.578	0.549	0.582	0.599	0.501	0.839	
DC	0.764	0.715	0.732	0.644	0.549	0.671	0.799

AVE, average variance extracted on diagonal.

### Data analysis

We selected SmartPLS 3 as the test tool for the research model to verify its collinearity, path relationships, model interpretation ability, and prediction ability of the structural equation model, and to improve and modify the model according to the verification results.

Collinearity means that there is a high correlation between independent variables, which leads to unreliable prediction results of the model. When the model has serious collinearity, it will lead to a deviation in the statistical significance of the results. Generally, if the variance inflation factor (VIF) value of each variable is <10, then the variables of the model do not exhibit collinearity. The results of this test are shown in Table 6. The VIF of each variable is <10, which eliminates the possibility of multicollinearity.

**Table 6:** Collinearity diagnostics.

**Measurement items**	**VIF**
IK1	4.318
IK2	3.927
IK3	4.467
IK4	5.476
EK1	4.826
EK2	4.830
EK3	4.613
EK4	5.965
SA1	4.865
SA2	4.673
SA3	3.708
TM1	3.791
TM2	4.750
TM3	3.465
ED1	4.578
ED2	6.803
ED3	7.007
IE1	6.665
IE2	7.761
IE3	4.436
DC1	3.065
DC2	3.981
DC3	3.622
DC4	5.781

VIF, variance inflation factor; IK, internal knowledge; EK, external knowledge; SA, student analysis ability; TM, teaching management ability; ED, educational decision-making ability; IE, instructional evaluation ability; DC, data consciousness.

#### Path analysis

**Figure 2:**
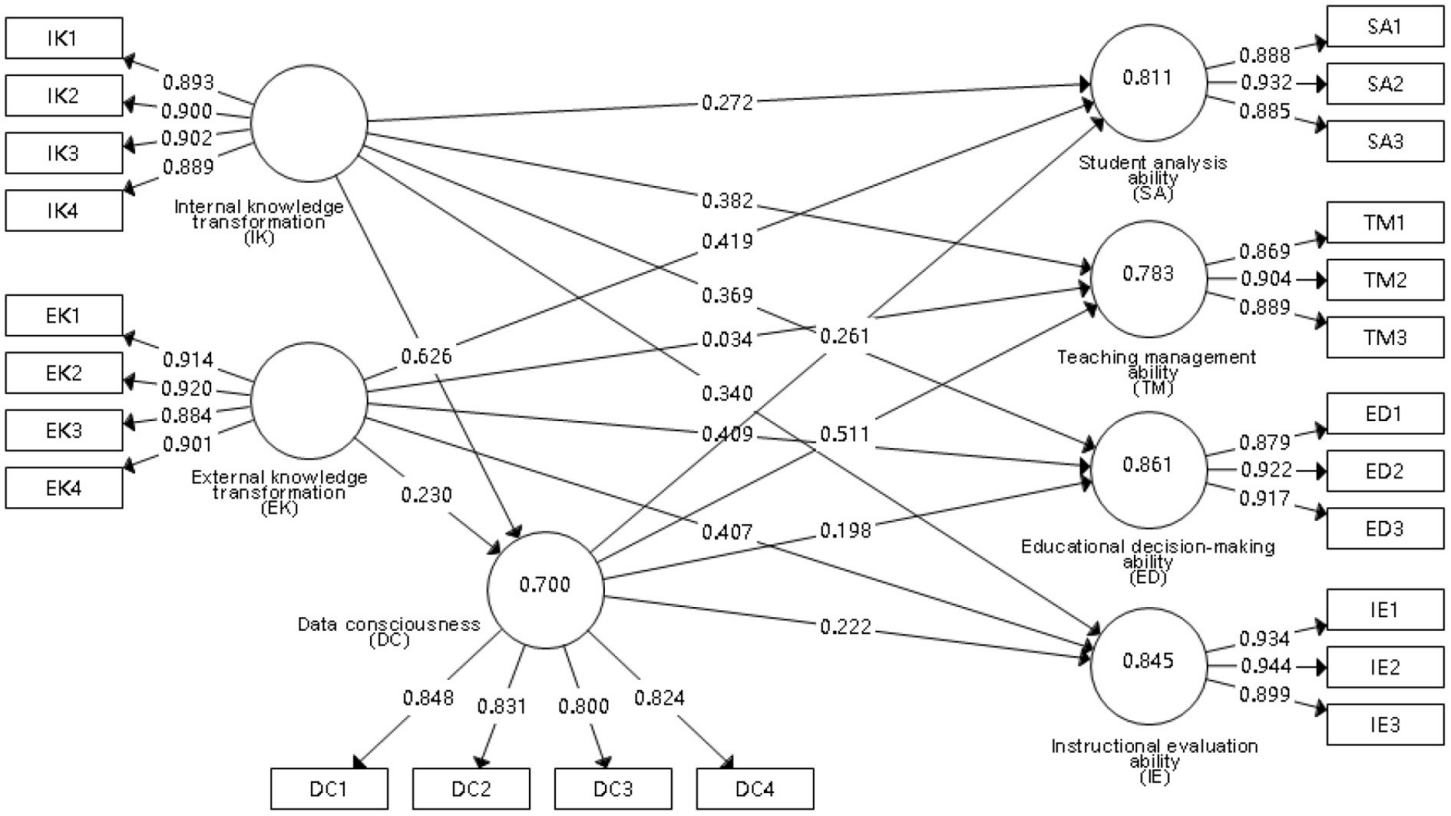
Path coefficient diagram.

Path analysis is a statistical method used to verify the causal relationship between multiple variables and its strength to obtain the accuracy and reliability of the causal model (Bernacki et al., 2015). The path analysis results for the variables obtained through the PLS Algorithm in SmartPLS 3 are shown in Figure 2. In the figure, IK refers to internal knowledge transformation, EK refers to external knowledge transformation, and DC refers to data consciousness. Teachers' precision teaching ability includes four potential variables: student analysis ability (SA), teaching management ability (TM), educational decision-making ability (ED), and instructional evaluation ability (IE).

The direct effects between factors are shown in Table 7. We find that H2b [External knowledge transformation has a positive impact on teaching management ability (EK → TM)] is not supported, while the other hypotheses are supported. The transformation of internal knowledge related to data has a significant positive impact on teachers' precision teaching ability and data consciousness, while the transformation of external knowledge related to teaching has no such impact on teachers' teaching management ability in precision teaching. Both internal knowledge transformation and external knowledge transformation have a significant positive impact on data consciousness, and data consciousness has a positive impact on the improvement of teachers' precision teaching ability.

**Table 7:** Verification of direct effect path relationships.

**Assumption**	**Relationship**	**Path coefficient**	***t*-value**	***p*-value**	**Decision**
H5c	DC → ED	0.198	3.950	0.000	Supported
H5d	DC → IE	0.222	3.897	0.000	Supported
H5a	DC → SA	0.261	4.277	0.000	Supported
H5b	DC → TM	0.511	7.264	0.000	Supported
H4	EK → DC	0.230	2.716	0.007	Supported
H2c	EK → ED	0.409	5.704	0.000	Supported
H2d	EK → IE	0.407	5.306	0.000	Supported
H2a	EK → SA	0.419	5.355	0.000	Supported
H2b	EK → TM	0.034	0.380	0.704	Unsupported
H3	IK → DC	0.626	7.562	0.000	Supported
H1c	IK → ED	0.369	4.366	0.000	Supported
H1d	IK → IE	0.340	3.880	0.000	Supported
H1a	IK → SA	0.272	3.007	0.003	Supported
H1b	IK → TM	0.382	3.995	0.000	Supported

IK, internal knowledge; EK, external knowledge; SA, student analysis ability; TM, teaching management ability; ED, educational decision-making ability; IE, instructional evaluation ability; DC, data consciousness.

The results of the indirect effect path relationship are shown in Table 8, which indicates that the indirect path relationship decisions of the factors are valid. Specifically, there is an indirect relationship between internal knowledge transformation, external knowledge transformation, data consciousness, and teachers' precision teaching ability. To illustrate the mediating effect of data consciousness, this effect is evaluated based on the indirect path relationship established in Table 8. The overall effect between the factors is calculated by the sum of the path coefficients of the direct effect and the indirect effect. However, the significance of the indirect path relationship cannot directly reflect the intermediary effect of the factors, and the variant allele fraction (VAF) of factors should be considered. Generally, if the VAF is <20%, the factor has no intermediary effect; if it is between 20 and 80%, it has some intermediate effect; and if it is >80%, it has a complete mediating effect. The results for the overall effect and VAF are shown in Table 9.

**Table 8:** Verification of the indirect path relationships.

**Relationship**	**Path coefficient**	***t*-value**	***p*-value**	**Decision**
EK → DC → TM	0.117	2.368	0.018	Supported
IK → DC → SA	0.163	3.401	0.001	Supported
EK → DC → ED	0.045	2.275	0.023	Supported
IK → DC → ED	0.124	3.149	0.002	Supported
EK → DC → SA	0.060	2.291	0.022	Supported
IK → DC → TM	0.320	5.187	0.000	Supported
EK → DC → IE	0.051	2.032	0.043	Supported
IK → DC → IE	0.139	3.389	0.001	Supported

IK, internal knowledge; EK, external knowledge; SA, student analysis ability; TM, teaching management ability; ED, educational decision-making ability; IE, instructional evaluation ability; DC, data consciousness.

**Table 9:** Evaluation of the mediation effect.

**Relationship**	**Direct effect**	**Indirect effect**	**Overall effect**	**VAF**	**Intermediary effect**
EK → TM	0.034	0.117	0.151	77.5%	Part
IK → SA	0.272	0.163	0.435	37.5%	Part
EK → ED	0.409	0.045	0.454	9.9%	Null
IK → ED	0.369	0.124	0.493	25.2%	Part
EK → SA	0.419	0.060	0.479	12.5%	Null
IK → TM	0.382	0.320	0.702	45.6%	Part
EK → IE	0.407	0.051	0.458	11.1%	Null
IK → IE	0.340	0.139	0.479	29.0%	Part

VAF, variant allele fraction; IK, internal knowledge; EK, external knowledge; SA, student analysis ability; TM, teaching management ability; ED, educational decision-making ability; IE, instructional evaluation ability; DC, data consciousness.

#### Evaluation of model detection and prediction ability

Table 10 displays the results for the model's interpretation and prediction ability; the explanatory power of the model includes the explanatory effect of the exogenous variables and endogenous variables of the model. Among them, the interpretation of the effect of the exogenous variables on the endogenous variables is determined by *f*^2^, and the effect of the endogenous variables is determined by the coefficient *R*^2^. When *f*^2^ is <0.15, the explanatory power of the exogenous variables is weak; when it is >0.15 and <0.35, it is moderate; when it is >0.35, it is strong. As shown in Table 10, the explanatory effect of the exogenous variables in this model is mainly between 0.15 and 0.35, with moderate explanatory power. In this study, the coefficient of determination is around 0.75; thus, the explanatory power of the endogenous variables is relatively strong.

**Table 10:** Verification of the explanatory and predictive abilities of the model.

**Relationship**	***f*** ^2^	***R*** ^2^	***Q*** ^2^
DC → ED	0.084	0.861	0.847
EK → ED	0.240		
IK → ED	0.159		
DC → IE	0.095	0.845	0.827
EK → IE	0.211		
IK → IE	0.121		
DC → SA	0.108	0.811	0.786
EK → SA	0.184		
IK → SA	0.063		
DC → TM	0.360	0.783	0.698
EK → TM	0.001		
IK → TM	0.109		
EK → DC	0.036	0.700	0.694
IK → DC	0.268		

IK, internal knowledge; EK, external knowledge; SA, student analysis ability; TM, teaching management ability; ED, educational decision-making ability; IE, instructional evaluation ability; DC, data consciousness.

Finally, the predictive ability of the model is measured by the value of *Q*^2^ obtained by blind-folding. If the values of *Q*^2^ are >0, the model has the ability to predict the endogenous variables; the larger the value, the stronger the predictive ability of the model. The values of *Q*^2^ in this model are high, so the predictive ability is significant.

Based on the comprehensive analysis, the interpretation and prediction capabilities of this model are relatively significant.

#### Model updating

**Figure 3:**
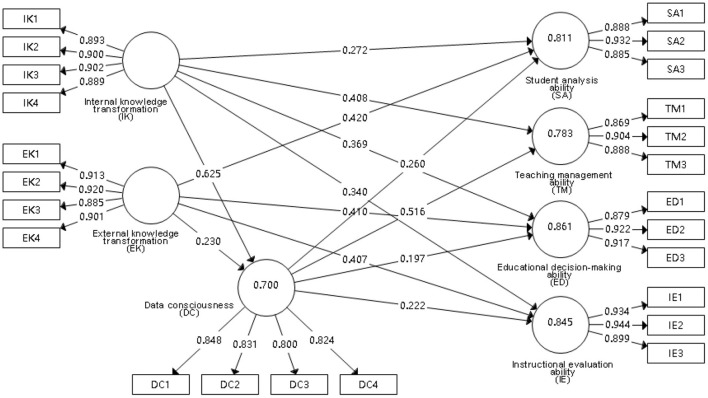
Revised theoretical model and path coefficient.

To verify the path relationship, the direct effect assumes that EK → TM fails the significance test, so it is deleted from the model. The modified model is shown in Figure 3.

In testing the intermediary effects, we assume that data consciousness is not an intermediary variable in the path: EK → DC → ED, EK → DC → SA, EK → DC → IE. On this basis, the mediating effects of the modified theoretical model were checked, as shown in Table 11.

**Table 11:** Revised mediation effect verification.

**Assumption**	**Relationship**	**VAF**	**Intermediary effect**	**Decision**
H6a	IK → DC → SA	27%	Part	Supported
H6b	IK → DC → TM	44%	Part	Supported
H6c	IK → DC → ED	20%	Part	Supported
H6d	IK → DC → IE	23%	Part	Supported
H6f	EK → DC → TM	100%	Complete	Supported

VAF, variant allele fraction; IK, internal knowledge; EK, external knowledge; SA, student analysis ability; TM, teaching management ability; ED, educational decision-making ability; IE, instructional evaluation ability; DC, data consciousness.

## Discussion

Most of the existing studies have concentrated on analyzing the definition of teachers' precision teaching ability or strategies to enhance them. However, the different roles that explicit and invisible data knowledge learning and transformation can play in enhancing teachers' precision teaching ability and the interrelationships between them remain to be clarified. Therefore, this study constructs a mediating effect model between data knowledge transformation and teachers' precision teaching competencies based on knowledge management theory.

The results of this study suggest that data knowledge learning and transformation play an important role in the improvement of teachers' precision teaching ability, and also reveal some important differences.

Regarding the impact of teachers' learning and transformation of data knowledge on improving their teachers' precision teaching ability (RQ1), our findings add to the literature. The learning and transformation of data knowledge can be divided into two categories: internal knowledge (related to data) and external knowledge (related to teaching). Among them, the former has a positive impact on teachers' precision teaching ability. While the latter has a positive effect on student analysis ability (SA), educational decision-making ability (ED), and instructional evaluation ability (IE), and has no significant positive correlation with the ability to manage teaching (TM).

The results of this study show that data consciousness plays a specific mediating effect between data knowledge transformation and teachers' precision teaching ability (RQ2). Among them, data consciousness partially mediates the effect between internal knowledge transformation (data-related) and teachers' precision teaching competencies. Between external knowledge transformation (teaching-related) and teachers' ability to teach accurately, data consciousness only fully mediated the effect between external knowledge transformation and instructional management ability.

In detail, the study found the following points. First, teachers' increased data consciousness relies on systematic learning of data knowledge. Teachers prefer activities such as smart education and precision teaching that revolve around knowledge of data collection, statistics, and analysis.

Second, teachers have a high level of recognition of data-related knowledge. For teachers, the learning and transformation of internal knowledge can effectively enhance their data consciousness, thus improving teachers' precision teaching ability.

Third, teachers pay less attention to training on teaching-related knowledge. Teachers generally believe that learning analysis, instructional decision making and instructional evaluation in data-driven teaching process are mainly influenced by educational experience, while data consciousness cannot be used as a mediating factor.

Finally, the group of instructional managers among teachers perceived data consciousness as having a significant mediating effect. They believe that both learning related to internal knowledge (data-related) and external knowledge (teaching-related) are needed at the instructional management level. Both of them work together to effectively enhance data consciousness and ultimately improve precision instructional management skills.

## Conclusion and implications

The findings of this study are of significant practical value as it provides indications for future targeted training of teachers in precision teaching competencies.

We need to understand the data confusion of teachers and focus on the development of teachers' precision teaching ability. At this stage, teachers' data confusion remains an important issue limiting the development of teachers' precision teaching ability. The research results show that teachers' confusion about the intrinsic knowledge of data (related to data) is much greater than the applied knowledge confusion of extrinsic knowledge (related to teaching). Teachers have many problems with the knowledge of data acquisition, statistics, organization, and analysis.

In this way, the activities focusing on the development of teachers' precision teaching ability should slow down the process of the activities. Before the activity is carried out, we should effectively understand the data confusion of the learning targets, focus on solving teachers' data problems first, and improve teachers' understanding of data value, data consciousness, and precision teaching. Learning about data as a way to enhance teachers' data confidence and reduce their sense of burden in precision teaching. Whether the learning activities related to precision teaching are organized by education departments or schools, the learning topics should be tailored to teachers' actual data confusion and provide appropriate learning materials.

At this stage, teachers' data confusion remains an important issue that restricts the development of teachers' precision teaching ability. Teachers as the executors of precision teaching, the implementation effect of precision teaching mainly depends on teachers' ability to understand, analyze and apply data. Whether the learning activities related to precision teaching are organized by education departments or schools, the learning topics should be tailored to teachers' actual data confusion and provide appropriate learning materials.

We must emphasize the development of data consciousness and optimize data-related knowledge translation mechanisms. The results of the study found that most teachers believe that data-related knowledge transformation can promote the development of data consciousness, which can ultimately enhance their own data literacy. The results of the study found that most teachers believe that data-related knowledge transformation can promote the development of data consciousness, which can ultimately enhance teachers' precision teaching ability.

Training activities should enhance the process of developing from technology to consciousness as teachers engage in internal knowledge of data-related platform development and setup, reading and computing, software use and processing, and data security. It should not be limited to text-based learning, but should focus on teachers' operational learning. Teachers should have a practical experience of data reliability, ease of operation, and its importance to teaching and learning during the training activities.

The learning of data technology knowledge can deepen teachers' understanding of data, reduce their sense of powerlessness and rejection of data, and promote the development of teachers' data consciousness. It allows teachers to understand the goal, process, and meaning of precision teaching at the cognitive level, thus enhancing their initiative to learn and apply data knowledge and optimize their transformation process.

In the training, we want to create realistic scenarios for application and focus on knowledge transformation mechanisms for data-driven teaching and learning processes. A large number of real teaching cases should be provided for the participants in the teachers' participation in data knowledge training, and virtual courses for accurate teaching and learning. To cope with the transformation of external knowledge (related to teaching), teachers not only need to be familiar with mastering data-related knowledge, but also need a lot of teaching and learning knowledge and experience in order to complete the transfer and transformation of knowledge. Create real scenario application, guide teachers to experience the process of data collection, collation, analysis, evaluation and prediction in a real environment, and feel the process and meaning of application in the process of learning. This is very essential.

Finally, we need to develop strategies for the development of group roles. Because different group roles require different focuses on teachers' precision teaching ability. Training should be designed and implemented to respond to different needs.

## Limitations and future research

Of course, there are still some shortcomings in this study, such as the limited sample size, insufficient diversity in the samples, the influence of subjective factors in the questionnaire design process, and the lack of qualitative data, such as observations and interviews. In subsequent research, we should increase the proportion of qualitative data collected and expand the sample size appropriately.

In addition, it is important to suggest future research directions for those interested in the unifying theme of this study. In the course of our research, we found that there are multiple groups of teachers, including teaching, management, and administration, and that the needs and perceptions of different groups differ significantly in terms of precision teaching competencies. It would be helpful if future research could delve into this study with specific categories of teacher groups.

## Data availability statement

The original contributions presented in the study are included in the article/supplementary material, further inquiries can be directed to the corresponding author.

## Ethics statement

Ethical review and approval was not required for the study involving human participants in accordance with the local legislation and institutional requirements. Written informed consent to participate in this study was not required from the participants in accordance with the national legislation and the institutional requirements.

## Author contributions

JT: project administration and data curation. CF: methodology and writing review and editing. WW: analysis data and perfect the thesis. YZ: revise the thesis and translation. All authors contributed to the article and approved the submitted version.
